# Nanomaterial-based cancer immunotherapy: enhancing treatment strategies

**DOI:** 10.3389/fchem.2024.1492215

**Published:** 2024-10-10

**Authors:** Mengxiang Tian, Xionglin Liu, Haiping Pei

**Affiliations:** ^1^ Department of Hepatobiliary Surgery, Guangxi Medical University Cancer Hospital, Nanning, Guangxi, China; ^2^ Department of General Surgery, Xiangya Hospital, Central South University, Changsha, China; ^3^ Guangxi Key Laboratory for High-Incidence Tumor Prevention and Treatment, Guangxi Medical University, Nanning, Guangxi, China

**Keywords:** nanomaterials, cancer immunotherapy, drug delivery, precision targeting, CAR-T therapy

## Abstract

Cancer immunotherapy has emerged as a pivotal approach for treating various types of cancer, incorporating strategies such as chimeric antigen receptor T-cell (CAR-T) therapy, immune checkpoint blockade therapy, neoantigen peptides, mRNA vaccines, and small molecule modulators. However, the clinical efficacy of these therapies is frequently constrained by significant adverse effects and limited therapeutic outcomes. In recent years, the integration of nanotechnology into cancer immunotherapy has gained considerable attention, showcasing notable advantages in drug delivery, targeted accumulation, controlled release, and localized administration. This review focuses on nanomaterial-based immunotherapeutic strategies, particularly the development and application of nanocarriers such as liposomes, lipid nanoparticles, polymeric nanoparticles, and self-assembling scaffolds. We examine how these strategies can enhance the efficacy of cancer immunotherapy while minimizing adverse effects and analyze their potential for clinical translation.

## Introduction

Cancer is indeed a major social, public health, and economic issue of the 21st century. According to the Global Cancer Statistics 2022 report (GLOBOCAN 2022) released by the International Agency for Research on Cancer (IARC), nearly 20 million new cancer cases were diagnosed worldwide in 2022, with cancer-related deaths reaching 9.7 million, making it the second leading cause of death after cardiovascular diseases (Bray et al., 2024). This increasing trend poses a significant challenge to global healthcare systems, particularly in low- and middle-income countries with limited medical resources (Shelton et al., 2024). Cancer immunotherapy offers an innovative approach to cancer treatment by activating or enhancing the patient’s immune system to mount an anti-tumor response (Zhang and Zhang, 2020). In recent years, with a more profound understanding of the interactions between the immune system and tumors, immunotherapy has rapidly evolved into a leading area of research in cancer treatment. Traditional therapeutic modalities such as surgery, chemotherapy, and radiotherapy have been foundational in cancer management but often lack specificity and are associated with a high risk of adverse effects (Borgers et al., 2021). In contrast, immunotherapy aims to specifically recognize and target tumor cells by reactivating the body’s natural defense mechanisms, thereby providing higher targeting precision and potential long-term protection (Yu et al., 2019).

Currently, a variety of immunotherapeutic strategies, including CAR-T cell therapy, immune checkpoint blockade therapy, personalized vaccines (such as neoantigen peptides and mRNA vaccines), and small molecule immunomodulators, have demonstrated varying levels of clinical efficacy across multiple cancer types (Yang et al., 2023). For instance, CAR-T cell therapy has achieved remarkable success in certain hematologic malignancies, such as acute lymphoblastic leukemia, while immune checkpoint blockade therapies (e.g., PD-1/PD-L1 inhibitors) have shown significant anti-tumor activity in several solid tumors (Zheng et al., 2024; Morad et al., 2021). Nevertheless, despite the substantial therapeutic potential of these approaches, they face several challenges in clinical application, including uneven drug distribution, systemic toxicity, inhibitory effects of the tumor microenvironment, and immune evasion (Sterner and Sterner, 2021). These challenges significantly limit the clinical applicability and efficacy of immunotherapies.

To address these challenges, the advancement of nanotechnology offers new opportunities for cancer immunotherapy. Nanomaterials, due to their unique physicochemical properties, have shown considerable potential in enhancing drug stability, biodistribution, and controlled release characteristics. These nanoscale carriers not only protect therapeutic agents from degradation within the body but also improve drug bioavailability, stability, and circulation time, thereby enabling more effective drug delivery (Xu et al., 2023). Moreover, nanomaterials can achieve precise drug release in response to the specific microenvironment of tumors, maximizing therapeutic efficacy while minimizing damage to normal tissues. This ability to provide targeted delivery and localized release underscores the significant advantages of nanomaterials in reducing systemic toxicity and enhancing therapeutic targeting, particularly in immunotherapies that require high drug doses or prolonged exposure (Pei et al., 2022). By improving tumor targeting, nanomaterials can markedly reduce systemic adverse effects. Current research indicates that nanomaterials can optimize the outcomes of various immunotherapies. The design and application of nanomaterials have broadened the scope of immunotherapy and provided new tools for achieving personalized and precise cancer treatment (Peng et al., 2022). Therefore, exploring and advancing nanomaterial-based immunotherapy strategies has become a crucial direction for improving cancer treatment outcomes, reducing adverse side effects, and facilitating clinical translation.

### Overview of cancer immunotherapy

**FIGURE 1:**
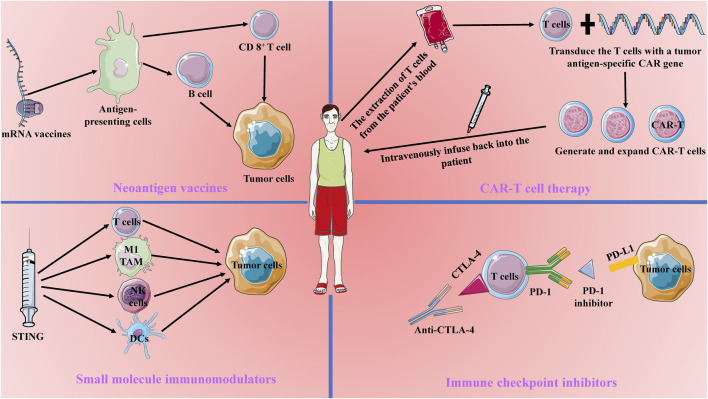
Current Main Approaches in Tumor Immunotherapy. Current tumor immunotherapy approaches include Chimeric Antigen Receptor (CAR) T-cell therapy, immune checkpoint blockade therapy, mRNA vaccines, and small molecule modulators. CAR-T therapy involves transducing patient-derived T cells with a tumor antigen-specific CAR gene and reinfusing them into the patient to enhance the T cells’ tumor-specific antitumor capabilities. Immune checkpoint blockade therapy works by blocking CTLA-4 and PD-1 to counteract tumor immune evasion. mRNA vaccines target antigen-presenting cells to activate B cells and T cells, thereby enhancing long-lasting antitumor immunity. Small molecule modulators, such as STING, activate the tumor immune microenvironment, promoting the killing of tumor cells by dendritic cells, NK cells, M1-type tumor-associated macrophages, and CD8+ T cells.

Cancer immunotherapy has become a cornerstone in modern cancer treatment by mobilizing and enhancing the patient’s immune system’s capacity to fight tumors. Compared to traditional treatments like surgery, chemotherapy, and radiotherapy, immunotherapy has garnered significant attention for its strong specificity and potential for durable anti-tumor effects (Llovet et al., 2024). The core principle of cancer immunotherapy lies in activating or modulating the patient’s immune system to recognize and attack tumor cells. Current immunotherapeutic strategies primarily include CAR-T cell therapy, immune checkpoint inhibitors, neoantigen vaccines, and small molecule immunomodulators. These approaches employ different mechanisms to overcome the inhibitory effects of the tumor microenvironment and restore the immune system’s anti-tumor activity (Adams et al., 2024) (Figure 1).

CAR-T cell therapy is a personalized treatment strategy that involves genetically engineering a patient’s T cells. By extracting T cells from the patient and modifying them in the laboratory to express a specific chimeric antigen receptor (CAR), these engineered T cells can more effectively recognize and kill tumor cells (Dagher and Posey, 2023). CAR-T therapy has achieved revolutionary progress in hematologic malignancies, such as acute lymphoblastic leukemia, and has demonstrated long-lasting anti-tumor effects in these diseases. However, its application in solid tumors remains limited due to the complexity of the solid tumor microenvironment, barriers to T cell infiltration, and tumor antigen heterogeneity (Yan et al., 2023). Additionally, despite its remarkable efficacy in certain hematologic malignancies, CAR-T cell therapy is associated with severe side effects, such as cytokine release syndrome (CRS) and neurotoxicity, which can lead to serious complications and even life-threatening conditions (Freyer and Porter, 2020).

Immune checkpoint inhibitors, such as PD-1/PD-L1 and CTLA-4 inhibitors, function by blocking immunosuppressive signals and alleviating the negative regulation of T cells, thereby restoring their anti-tumor activity (Bagchi et al., 2021). These agents have shown significant efficacy in various cancers, including melanoma and non-small cell lung cancer, and have become standard treatment options for these malignancies. However, not all patients respond to immune checkpoint inhibitors, with only about 25% demonstrating a significant response, while others may show no response or develop early resistance. This variability in efficacy makes it challenging for immune checkpoint inhibitors to serve as a standalone treatment (Ghiringhelli et al., 2023; Lurienne et al., 2020; Park et al., 2023). Moreover, while these inhibitors can “release the brakes” on the immune system, they may also cause excessive immune activation, leading to autoimmune diseases such as dermatitis, colitis, and endocrine dysfunctions. In some cases, these immune-related adverse events may require long-term immunosuppressive treatment, adversely affecting patients’ quality of life (Viscardi et al., 2022).

Neoantigen vaccines represent another promising immunotherapy strategy, designed to elicit robust and durable immune responses by targeting tumor-specific mutations (Katsikis et al., 2024). Current research focuses on neoantigen peptide vaccines and mRNA vaccines. Neoantigen peptide vaccines directly activate T cells by synthesizing peptide segments, while mRNA vaccines induce an immune response by translating and expressing neoantigens *in vivo* (Rojas et al., 2023). Although these vaccines are highly personalized in design and preparation, their efficacy is still limited by tumor antigen heterogeneity and immune evasion mechanisms (Hu et al., 2021; Lorentzen et al., 2022).

Small molecule immunomodulators enhance the immune system’s anti-tumor capacity by interfering with specific immune signaling pathways, such as STING (Kwon and Bakhoum, 2020). These small molecules can directly affect immune cells or the tumor microenvironment to regulate immune responses. However, due to their rapid metabolism and uneven distribution in the body, the clinical efficacy of small molecule immunomodulators is often limited (Samson and Ablasser, 2022).

In conclusion, while immunotherapy has demonstrated tremendous potential in cancer treatment, its existing limitations and associated side effects necessitate further research and development. To overcome these challenges, leveraging nanomaterials to address the current shortcomings of immunotherapies offers promising avenues for providing effective treatment options to a broader range of cancer patients (Lu et al., 2024) (Table 1).

**TABLE 1:** Characteristics and limitations of cancer immunotherapy.

Therapeutic approaches	Mechanism	Characteristics	Limitations
CAR-T cell therapy	Modify the patient’s T cells to more effectively recognize and destroy tumor cells	Demonstrated durable antitumor effects in hematologic malignancies	Poor efficacy in solid tumors and severe toxic side effects
Immune checkpoint inhibitors	Block immunosuppressive signaling pathways	Significant efficacy in treating certain tumors	Variability in efficacy and the occurrence of immune overactivation
Neoantigen vaccines	Directly activate T cells through the synthesis of peptide fragments	Elicit a strong, durable, and specific immune response	Efficacy is still limited by tumor antigen heterogeneity and immune evasion mechanisms
Small molecule immunomodulators	Directly act on the tumor microenvironment	Directly enhance the sensitivity of the tumor microenvironment to the immune system	Uneven distribution in the body and rapid metabolism

### Advantages of nanomaterials in tumor therapy

In the realm of cancer immunotherapy, the effectiveness and precision of drug delivery are critical to achieving favorable therapeutic outcomes. Traditional drug delivery methods, while effective in certain cases, often face challenges such as poor drug stability, inadequate targeting, and significant systemic toxicity. These limitations can reduce the overall efficacy of treatment and adversely affect patients’ quality of life (Ashrafizadeh et al., 2023). Nanomaterials, as an emerging drug delivery platform, possess unique physicochemical properties that can effectively address these challenges.

Liposomes are nanoscale particles composed of a phospholipid bilayer, offering excellent biocompatibility and biodegradability. Their aqueous core can encapsulate hydrophilic drugs, while the lipid bilayer can incorporate lipophilic drugs, making liposomes highly versatile as drug carriers (Liu et al., 2022). Liposomes can enhance drug stability; many anticancer drugs are prone to degradation by metabolic enzymes or rapid clearance from the bloodstream, leading to reduced effective drug concentrations. Encapsulating drugs within liposomes can significantly extend their half-life in the body, thereby improving bioavailability (Gu et al., 2023). Another major advantage of liposomes is their potential for controlled release. By adjusting the composition and surface properties of liposomes, carriers with different release rates can be designed. For instance, temperature-sensitive or pH-sensitive liposomes can achieve rapid drug release under specific physiological conditions (Ding et al., 2022). This controlled release capability is particularly important in cancer immunotherapy, as it ensures that drugs exert their effects at the optimal time and location, thereby improving therapeutic outcomes (Shields et al., 2020).

Lipid Nanoparticles (LNPs) are nanoscale particles composed of solid or liquid lipids, typically featuring a lipid core and an outer shell made of phospholipids or other lipid molecules. This structure grants LNPs excellent drug loading capacity and stability (Zong et al., 2023). Compared to liposomes, LNPs provide higher drug loading capacity and better physical stability due to their solid or semi-solid lipid cores, which allow drugs to be more effectively embedded, enhancing drug stability and loading efficiency (Estapé Senti et al., 2024). Additionally, the surface of LNPs can be modified with polyethylene glycol (PEG) or other hydrophobic molecules to extend their circulation time in the blood and reduce the likelihood of clearance by the mononuclear phagocyte system (Huang et al., 2023). LNPs also offer significant advantages in targeted delivery and localized release. By modifying the surface with targeting ligands, LNPs can specifically target tumor cells or particular molecules within the tumor microenvironment, enabling precise drug delivery. In the context of mRNA vaccine delivery, LNPs protect mRNA from degradation by nucleases within the body and facilitate its effective entry into target cells (Cheng et al., 2020; Kon et al., 2023). The successful application of LNPs in CRISPR-Cas9 has further validated their potential in nucleic acid drug delivery, laying a foundation for their use in cancer immunotherapy (Rosenblum et al., 2020).

Polymeric Nanoparticles (PNPs) are nanoscale particles made from natural or synthetic polymers, including structures such as solid nanoparticles, nanomicelles, or nanocapsules. Their greatest advantage lies in their highly tunable physicochemical properties. By selecting different polymer materials and synthesis methods, the particle size, shape, surface charge, and degradation rate of PNPs can be precisely controlled (Li et al., 2018; Rajana et al., 2022). For example, by incorporating stimulus-responsive polymers (e.g., pH, temperature, or redox-sensitive polymers), PNPs can trigger drug release in specific pathological environments, thereby enhancing treatment specificity and efficacy (Kim et al., 2024). For targeted delivery, PNPs can be surface-modified with multifunctional ligands to achieve targeted delivery to specific cells or tissues.

Self-Assembled Scaffolds are nanostructures formed spontaneously through intermolecular interactions, such as hydrogen bonding, electrostatic forces, or hydrophobic interactions. The structural and functional characteristics of self-assembled scaffolds provide significant advantages in controlled release and localized delivery. By adjusting the molecular design of self-assembled scaffolds, the release rate of drugs can be precisely controlled (Xu et al., 2022). For instance, using pH-sensitive self-assembled scaffolds, drug release can be triggered within the acidic tumor microenvironment, thereby increasing drug concentration and efficacy at the tumor site (Jafarzadeh-Holagh et al., 2018; Sun et al., 2021). Moreover, self-assembled scaffolds can be combined with immune checkpoint inhibitors or small molecule immunomodulators to achieve multi-target regulation in immunotherapy, thereby enhancing overall therapeutic efficacy (Hu et al., 2024; Ren et al., 2023).

In summary, the application of nanomaterials in tumor immunotherapy offers substantial advantages, including improved drug stability, enhanced targeting, localized delivery, controlled release, and reduced systemic toxicity. These benefits not only increase drug concentration and efficacy at the tumor site but also minimize damage to normal tissues, thereby reducing systemic side effects (Table 2).

**TABLE 2:** A summary of the characteristics of nanomaterials in cancer therapy.

Classification	Material properties	Advantages	Address which shortcomings in immunotherapeutic agents
Liposomes	The membrane-like structure facilitates drug encapsulation	Both water-soluble and lipid-soluble drugs can be encapsulated	Extend drug half-life, enhance targeting, and control drug release
Lipid Nanoparticles	Consist of solid or liquid lipids to form a stable structure	Higher drug loading capacity and improved stability	Enhance drug half-life and targeted delivery
Polymeric Nanoparticles	Diverse structures of solid nanoparticles, nanomicelles, or nanocapsules	Highly tunable physicochemical properties	Control drug release and targeted delivery
Self-Assembled Scaffolds	Structures spontaneously formed through intermolecular interactions	Enhanced particle size control and improved ordering	Precise drug release and targeted delivery

### Application of nanomaterials in tumor immunotherapy

**FIGURE 2:**
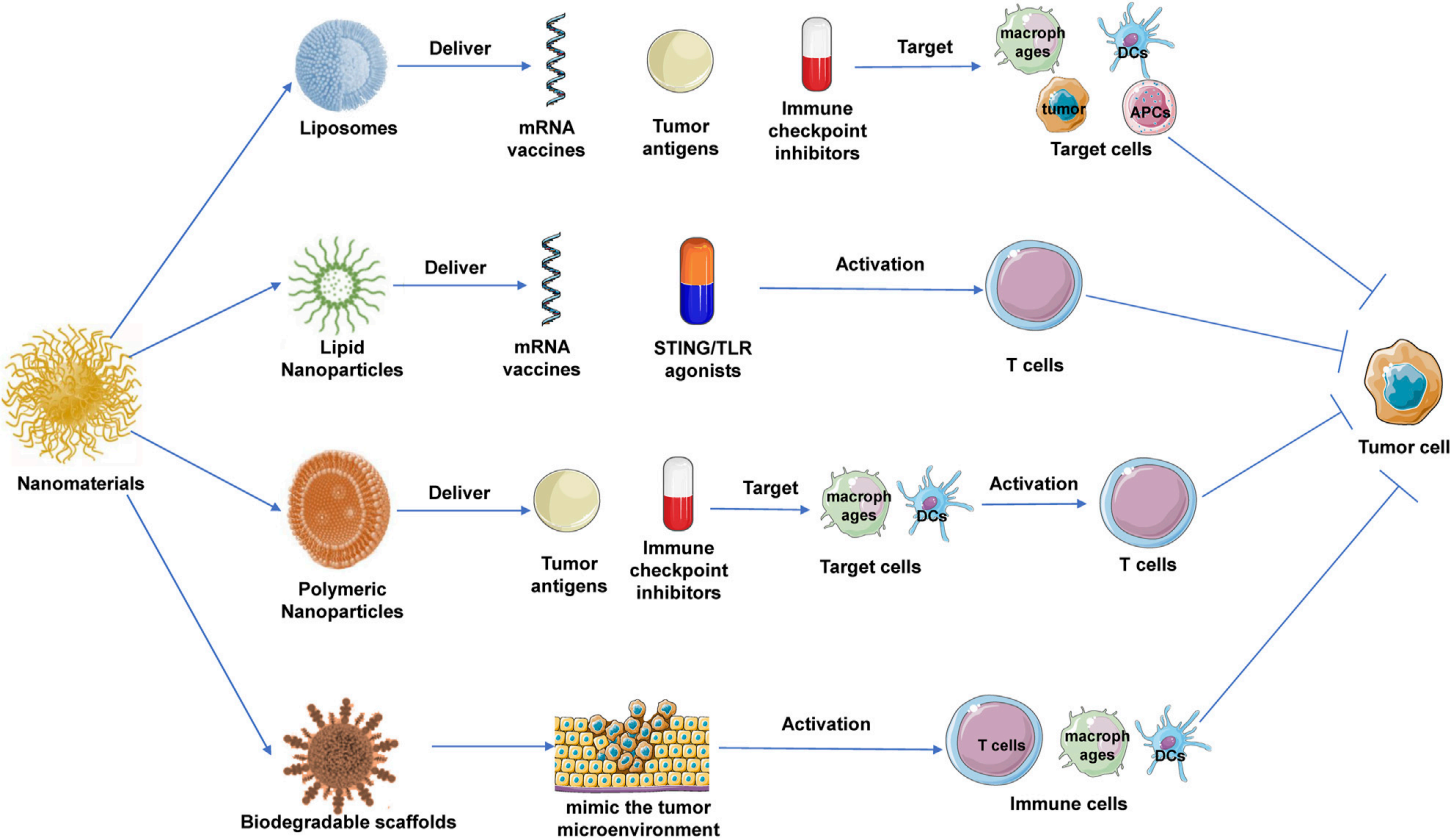
Application of nanomaterials in tumor immunotherapy. Liposomes, lipid nanoparticles (LNPs), and polymeric nanoparticles (PNPs) are commonly used nanomaterials in cancer immunotherapy. Their specific anti-tumor mechanisms include: liposomes, which enhance antigen presentation by targeting dendritic cells and can deliver immune checkpoint inhibitors; LNPs, which are used to deliver mRNA vaccines and immunomodulators, activating T cell responses; and PNPs, which target immune cells to deliver antigens or small-molecule modulators to promote tumor immune clearance. Additionally, degradable scaffolds provide a localized immune microenvironment for tumors.

The application of nanomaterials in cancer immunotherapy has emerged as a significant area of research in recent years. By encapsulating anticancer drugs, immune checkpoint inhibitors, antigenic peptides, and mRNA within nanoscale carriers, researchers can achieve more precise and effective drug delivery, modulating the tumor microenvironment to enhance the immune system’s antitumor capabilities (Figure 2).

Liposomes are among the most widely studied nanomaterials in tumor immunotherapy. They are commonly used to deliver antigens, which can improve the immune system’s ability to recognize and attack tumor cells. For example, encapsulating tumor antigens or neoantigen peptides within liposomes can enhance antigen stability and prolong their circulation time in the body, leading to more efficient antigen presentation (Chen et al., 2022). By modifying the surface of liposomes with antibodies or ligands, these nanocarriers can specifically target dendritic cells (DCs) or macrophages, which play crucial roles in antigen presentation and T-cell activation. Once dendritic cells uptake these liposomes, they can effectively process and present the antigens, inducing a strong T-cell response that further promotes tumor elimination (Liang et al., 2017). In addition to antigen delivery, liposomes are also employed to deliver immune checkpoint inhibitors, such as PD-1/PD-L1 and CTLA-4 inhibitors. Encapsulating these inhibitors within liposomes can increase their concentration within the tumor microenvironment, enhancing the effectiveness of immune checkpoint blockade (Ma and Lin, 2023). This localized delivery strategy reduces the distribution of immune checkpoint inhibitors in normal tissues, thereby minimizing autoimmune-related side effects. Furthermore, liposomes have made significant strides in vaccine delivery (Kiaie et al., 2022). For instance, encapsulating mRNA vaccines within liposomes can protect the mRNA from degradation by nucleases in the body and facilitate its effective delivery to target cells. Once inside the cells, the mRNA can be translated to express tumor antigens, inducing a specific T-cell response that enhances immune surveillance and attacks the tumor (Zong et al., 2023).

In cancer immunotherapy, LNPs have been widely used for delivering mRNA vaccines that encode specific tumor antigens, thereby activating the immune system to recognize and attack tumor cells (Kon et al., 2023). The successful application of LNPs in mRNA vaccine delivery is attributed to several unique advantages. Firstly, LNPs effectively protect mRNA from degradation within the body and facilitate the delivery of mRNA into the cytoplasm through membrane fusion (Wang et al., 2023). Once in the cytoplasm, the mRNA is translated into tumor antigen proteins, which are subsequently presented to T cells via the major histocompatibility complex (MHC), thereby activating a specific T-cell response. This antigen-specific T-cell immune response is crucial for the effective elimination of tumor cells (Su et al., 2022). LNPs are also employed to deliver immune modulators to enhance immune responses within the tumor microenvironment. For example, encapsulating STING agonists within LNPs can increase their accumulation at the tumor site and enhance the production of type I interferons through activation of the STING pathway, thereby promoting an antitumor immune response (Khalifa et al., 2024; Nakamura et al., 2021; Qiu et al., 2024). Similarly, LNPs can be used to deliver TLR agonists or immune checkpoint inhibitors to enhance antigen presentation and T-cell activation, ultimately improving the overall efficacy of immunotherapy (Korzun et al., 2024).

PNPs play a critical role in tumor immunotherapy, particularly in the delivery of antigens. By encapsulating tumor antigens within PNPs, the stability of these antigens can be significantly enhanced, and their half-life in the body extended (Blakney et al., 2021). The surface of PNPs can be modified with specific ligands, such as antibodies or peptides, to target dendritic cells or macrophages, which are key players in antigen presentation and T-cell activation. Once dendritic cells uptake PNPs, they can efficiently process and present the antigens, thereby inducing a potent T-cell response and promoting the immune clearance of tumors (Ben-Akiva et al., 2023; Wu et al., 2023). Moreover, PNPs can also be used to deliver immune modulators. Encapsulating these modulators within PNPs increases their concentration within the tumor microenvironment and allows for controlled release, ensuring sustained immune modulation (Chen et al., 2021). This delivery strategy enhances the immune response within the tumor microenvironment, thereby improving the overall effectiveness of immunotherapy. For instance, encapsulating PD-1/PD-L1 inhibitors within PNPs can significantly increase their concentration at the tumor site, thereby enhancing immune checkpoint blockade efficacy and reducing systemic toxicity (Gao et al., 2023; Shen et al., 2021).

Biodegradable scaffolds, as three-dimensional structural materials, can mimic the tumor microenvironment and provide an optimal platform for the growth and activation of immune cells. When implanted at the tumor site, these scaffolds create a localized environment that continuously releases antigens and immune modulators, thereby significantly boosting the local immune response (Aguado et al., 2018). Furthermore, biodegradable scaffolds can serve as carriers for the direct delivery of modified or activated immune cells, such as CAR-T cells or dendritic cells, to the tumor site (Agarwalla et al., 2022; Chao et al., 2023; Tousley et al., 2023). These scaffolds not only protect the immune cells from damage during the delivery process but also support their proliferation and functional activity within the tumor microenvironment, thereby enhancing the efficacy of tumor immunotherapy (Zhang et al., 2024).

### The clinical translation prospects of nanomaterials

The clinical translation of nanomaterials in tumor immunotherapy holds substantial promise. Firstly, nanomaterials provide an optimal platform for the efficient delivery of tumor antigens. Through the design of specific nanocarriers, antigens can be precisely delivered to antigen-presenting cells (APCs) (Shi and Lammers, 2019). This targeted delivery not only enhances the immunogenicity of the antigens but also significantly boosts the activation of tumor-specific T cells, thereby improving the overall effectiveness of immunotherapy (Cheng et al., 2021). With the advancement of magnetic field control technology (Winkler et al., 2023), magnetic nanoparticles can induce NK cell infiltration into tumor sites, providing a low-toxicity new approach for nanomaterial-based therapies that activate immune cells (Jang et al., 2012). Secondly, nanomaterials exhibit exceptional performance in the delivery of immune checkpoint inhibitors. Due to their high drug-loading capacity and prolonged circulation time, nanomaterials enable the sustained release of drugs within the tumor microenvironment, which significantly enhances the antitumor efficacy of the inhibitors while reducing systemic toxicity (Bai et al., 2022). Furthermore, nanomaterials can integrate multiple therapeutic strategies into a multifunctional system, creating a composite therapeutic platform with synergistic effects. For instance, by combining antigen delivery, immune checkpoint blockade, and immune microenvironment modulation into a single nanoplatform, it is possible to activate multiple immune pathways simultaneously, resulting in a more robust antitumor response (Hamilton et al., 2023).

Despite these promising prospects, the clinical translation of nanomaterials still faces several challenges, including the need for large-scale drug production, comprehensive long-term *in vivo* safety evaluations, and addressing potential immunogenicity concerns. Future research must focus on optimizing the design of nanomaterials to balance complexity with clinical feasibility and improving the alignment of preclinical models with human physiological conditions to accelerate their clinical application in tumor immunotherapy (Younis et al., 2022).

## Conclusion

Nanomaterial-based cancer immunotherapy presents innovative strategies to enhance the efficacy of immunotherapy. With the advancement of nanotechnology, nanomaterials, characterized by their unique physicochemical properties—such as high surface area, tunable surface modifications, and biocompatibility—are increasingly becoming integral tools in tumor immunotherapy. These materials facilitate the precise delivery of antigens, immune checkpoint inhibitors, and other immunomodulatory agents, thereby effectively improving therapeutic outcomes and minimizing systemic side effects.

However, the clinical translation of nanomaterials still faces numerous challenges, including large-scale production of the drugs, long-term *in vivo* safety assessments, and potential immunogenic reactions. Therefore, future research must focus on developing more efficient preparation processes and establishing strict quality control systems—crucial steps in enhancing the feasibility of nanomaterials in clinical applications. Additionally, integrating artificial intelligence and machine learning technologies into intelligent design will help scientists predict and adjust the properties of nanomaterials to better meet clinical needs. Lastly, the development of more advanced *in vivo* and *in vitro* models, especially those closely mimicking human physiological conditions, will enable more precise predictions of nanomaterials’ responses in the human body. Combined with subsequent multi-center, interdisciplinary collaborative studies, these efforts will help address the various challenges in the clinical translation of nanomaterials, promoting their journey from the lab to clinical practice. With these measures, nanomaterial-based immunotherapy is expected to become a key approach in cancer treatment in the future.

## Abbreviations

CAR-T, chimeric antigen receptor T-cell; LNPs, Lipid Nanoparticles; PNPs, Polymeric Nanoparticles; DCs, dendritic cells; MHC, major histocompatibility complex.
